# An Environmental Uncertainty Perception Framework for Misinformation Detection and Spread Prediction in the COVID-19 Pandemic: Artificial Intelligence Approach

**DOI:** 10.2196/47240

**Published:** 2024-01-29

**Authors:** Jiahui Lu, Huibin Zhang, Yi Xiao, Yingyu Wang

**Affiliations:** 1 State Key Laboratory of Communication Content Cognition People's Daily Online Beijing China; 2 School of New Media and Communication Tianjin University Tianjin China

**Keywords:** misinformation detection, misinformation spread prediction, uncertainty, COVID-19, information environment

## Abstract

**Background:**

Amidst the COVID-19 pandemic, misinformation on social media has posed significant threats to public health. Detecting and predicting the spread of misinformation are crucial for mitigating its adverse effects. However, prevailing frameworks for these tasks have predominantly focused on post-level signals of misinformation, neglecting features of the broader information environment where misinformation originates and proliferates.

**Objective:**

This study aims to create a novel framework that integrates the uncertainty of the information environment into misinformation features, with the goal of enhancing the model’s accuracy in tasks such as misinformation detection and predicting the scale of dissemination. The objective is to provide better support for online governance efforts during health crises.

**Methods:**

In this study, we embraced uncertainty features within the information environment and introduced a novel Environmental Uncertainty Perception (EUP) framework for the detection of misinformation and the prediction of its spread on social media. The framework encompasses uncertainty at 4 scales of the information environment: physical environment, macro-media environment, micro-communicative environment, and message framing. We assessed the effectiveness of the EUP using real-world COVID-19 misinformation data sets.

**Results:**

The experimental results demonstrated that the EUP alone achieved notably good performance, with detection accuracy at 0.753 and prediction accuracy at 0.71. These results were comparable to state-of-the-art baseline models such as bidirectional long short-term memory (BiLSTM; detection accuracy 0.733 and prediction accuracy 0.707) and bidirectional encoder representations from transformers (BERT; detection accuracy 0.755 and prediction accuracy 0.728). Additionally, when the baseline models collaborated with the EUP, they exhibited improved accuracy by an average of 1.98% for the misinformation detection and 2.4% for spread-prediction tasks. On unbalanced data sets, the EUP yielded relative improvements of 21.5% and 5.7% in macro-F1-score and area under the curve, respectively.

**Conclusions:**

This study makes a significant contribution to the literature by recognizing uncertainty features within information environments as a crucial factor for improving misinformation detection and spread-prediction algorithms during the pandemic. The research elaborates on the complexities of uncertain information environments for misinformation across 4 distinct scales, including the physical environment, macro-media environment, micro-communicative environment, and message framing. The findings underscore the effectiveness of incorporating uncertainty into misinformation detection and spread prediction, providing an interdisciplinary and easily implementable framework for the field.

## Introduction

### Background

The World Health Organization and the United Nations have issued warnings about an “infodemic,” highlighting the spread of misinformation alongside the COVID-19 pandemic on social media [1]. Misinformation is characterized as “factually incorrect information not backed up by evidence” [2]. This misleading information frequently encompasses harmful health advice, misinterpretations of government control measures and emerging sciences, and conspiracy theories [3]. This phenomenon has inflicted detrimental impacts on public health, carrying “severe consequences with regard to people’s quality of life and even their risk of mortality” [4].

Automatic algorithms are increasingly recognized as valuable tools in mitigating the harm caused by misinformation. These techniques can rapidly identify misinformation, predict its spread, and have demonstrated commendable performance. The state-of-the-art detection techniques exhibit accuracy ranging from 65% to 90% [5,6], while spread-prediction techniques achieve performance levels between 62.5% and 77.21% [7,8]. The high accuracy of these techniques can be largely attributed to the incorporation of handcrafted or deep-learned linguistic and social features associated with misinformation [9-11]. Scholars have consistently invested efforts in integrating theoretically relevant features into algorithmic frameworks to enhance accuracy further.

Scholars have introduced diverse frameworks for misinformation detection and spread-prediction algorithms. Nevertheless, existing frameworks have predominantly concentrated on the intricate post-level signals of misinformation, emphasizing linguistic and social features (such as user relationships, replies, and knowledge sources) associated with misinformation. Notably, these frameworks have often overlooked the characteristics of the information environment in which misinformation originates and proliferates [12]. This neglect could potentially result in diminished performance for misinformation detectors when applied in various real-world misinformation contexts. This is due to the fact that different misinformation contexts possess unique characteristics within their information environment, influencing the types of misinformation that can emerge and thrive [13]. An indispensable characteristic of the information environment concerning misinformation is uncertainty. Uncertainty arises when the details of situations are ambiguous, complex, unpredictable, or probabilistic, and when information is either unavailable or inconsistent [14]. In uncertain situations, individuals tend to generate and disseminate misinformation as a means of resisting uncertainty and seeking understanding amid chaotic circumstances [15,16]. The COVID-19 pandemic serves as a notable example, marked by a lack of understanding of emerging science [17], uncertainties surrounding official guidelines and news reports [18], and unknown impacts on individuals and society [19]. Hence, in this study, we recognize uncertainty as the pivotal feature in the information environment of misinformation. Our objective is to formulate a novel framework for perceiving environmental uncertainty, specifically tailored for the detection and spread prediction of misinformation during the COVID-19 pandemic.

Our contributions can be outlined as follows. Theoretically, we provide a comprehensive exploration of uncertainty across 4 distinct scales of the information environment, namely, the physical environment, macro-media environment, micro-communicative environment, and message framing. These scales collectively contribute to the emergence and dissemination of misinformation. Furthermore, we hold the distinction of being the pioneers in integrating Environmental Uncertainty Perception (EUP) into the realms of misinformation detection and spread prediction. In terms of methodology, we introduce the EUP framework, designed to capture uncertainty signals from the information environment of a given post for both misinformation detection and spread prediction. Our experiments conducted on real-life data underscore the effectiveness of the EUP framework.

This paper unfolds as follows: In the “Related Work” section, we provide a concise review of the related work. The “Proposed Theoretical Framework” section elucidates uncertainty features within the information environment, which are pertinent to misinformation detection and spread prediction. Moving on to the “Research Objectives” section, we outline our study objectives. The “Methods” section details our methodology for testing the proposed framework. In the “Data Set and Experiment” section, we present our data set, experiments, and comprehensive analyses. The “Discussion” section delves into discussions on our findings, unraveling the theoretical and practical implications of our work. Finally, the “Conclusions” section concludes with a summary and outlines directions for future research.

### Related Work

Detecting misinformation on social media represents a burgeoning research field that has garnered considerable academic attention. Multiple frameworks have been put forth for this task, primarily falling into 2 approaches: the post-only approach and the “zoom-in” approach [12]. In the former, frameworks focus on studying post features to differentiate misinformation from general information. Linguistic features, including novelty, complexity, emotions, and content topics, are frequently explored [6,11]. Additionally, researchers have delved into multimodal features, particularly those based on visuals [20,21]. Deep learning models in natural language processing have also proven beneficial for the misinformation detection task [5,22].

The “zoom-in” approach places emphasis on socio-contextual signals, centering on users’ networking aspects (eg, user relationships, number of replies, number of created threads; [23,24]) and network characteristics (eg, degree centrality [25]). Another line of research underscores the significance of relevant knowledge sources, including fact-checking websites [26] and knowledge graphs [27], which can be used to validate specific claims of interest.

Recently, Sheng et al [12] introduced a “zoom-out” approach, concentrating on the information environments of misinformation that can offer signals for detection. In their approach, they incorporated the news environment into fake news detection. Their hypothesis posited that fake news should not only be relevant but also novel and distinct from recent popular news, enabling them to capture audience attention and achieve widespread dissemination. Their findings revealed that signals of popularity and novelty can enhance the performance of state-of-the-art misinformation detectors.

In the realm of misinformation detection, misinformation spread prediction represents another challenging task, albeit one that has received limited attention. This task involves predicting whether a piece of misinformation is likely to be disseminated to a broader audience through actions such as likes, comments, and shares. Within this context, our specific focus is on predicting whether misinformation is likely to be retweeted. This can be viewed as a binary classification task, akin to misinformation detection. Frameworks for this task typically incorporate linguistic and social features, which may overlap with or differ from those used in misinformation detection. Linguistic features such as persuasive styles, emotional expressions, and message coherence prove valuable in predicting the spread of misinformation [28,29]. Additionally, social features, including user metadata (eg, number of friends, verification) and tweet metadata (eg, presence of images and URLs), are identified as relevant factors for predicting misinformation spread [25].

### Proposed Theoretical Framework

#### Uncertainty as a Central Aspect in Misinformation

Our study builds upon Sheng et al’s [12] “zoom-out” approach, adopting an interdisciplinary perspective that centers on the uncertainty within the information environment of misinformation. The realms of communication and psychology literature have conceptualized uncertainty as a fundamental aspect of misinformation. Uncertainty is said to prevail “when details of situations are ambiguous, complex, unpredictable, or probabilistic; uncertainty is also present when information is unavailable or inconsistent, and when individuals feel insecure about their own state of knowledge or the general state of knowledge” [14]. Confronted with uncertainty, individuals are driven to alleviate it by constructing their understanding of the situation [16]. This constructive process is known as sensemaking, which encompasses how individuals impart meaning to their surroundings and use it as a foundation for subsequent interpretation and action [30]. Sensemaking entails the utilization of information by individuals to fill gaps in their understanding [31]. Yet, the utilization of information in this manner does not always guarantee truth. In situations where information is slow to emerge, individuals are driven to comprehend uncertain situations by relying on their existing knowledge and heuristics for judgment. Unfortunately, this process often leads to the formation of false beliefs and misinformation [32]. Additionally, individuals may “turn to unofficial sources to satisfy their information needs,” potentially exposing themselves to inaccurate information [33]. As suggested by Kim et al [34], exposure to misinformation has the potential to diminish feelings of uncertainty. Moreover, as individuals integrate more information into their comprehension of a situation, there is a tendency to seek plausibility, which may lead to the generation and acceptance of misinformation [16,35].

The aforementioned tendencies are notably prominent in the context of the COVID-19 pandemic, as the pandemic represents a time of heightened uncertainty. The emergence of the pandemic was marked by a mysterious disease with previously unseen symptoms. Fundamental questions regarding the origins of the disease, measures for self-protection, and strategies for containing the outbreak were not immediately evident. As the pandemic progressed, uncertainty persisted regarding how and when the outbreak would be fully contained, as well as the long-term impact it would have on individuals and society. The uncertainty stemming from the pandemic, coupled with the surge of social media as a primary source of information, has facilitated the spread of misinformation [16].

Although many studies have identified “uncertainty” as a central aspect of misinformation, they have not thoroughly elucidated how uncertainty, as a crucial feature of the information environment, can aid in the detection of misinformation and the prediction of its spread. The literature frequently treats uncertainty as a static and holistic feature of a situation. However, the level of uncertainty within a situation can be dynamic, evolving as the situation progresses. For instance, uncertainties about the virus and the initial life changes induced by the COVID-19 pandemic would have been considerably higher at its onset than they are at present [36]. Moreover, uncertainty can manifest differently across various scales of the information environment. The information environment has become increasingly intricate with the proliferation of the internet and communication technologies. Individuals may be exposed to a substantial volume of information about trending topics through mainstream mass media (eg, newspapers, TV, social media trends) within a short time frame, constituting a macro-media environment. Simultaneously, they may selectively engage in detailed communications on a specific issue provided by self-media (eg, subscription accounts, self-broadcasting), shaping a micro-communicative environment. Uncertainty manifested in these 2 environments may independently or interactively influence people’s sensemaking processes and, consequently, their outputs (eg, misinformation). Additionally, uncertainty can be inherent in the misinformation itself, providing cues for its detection and spread prediction. We will elaborate on the features of uncertainty in the information environment in the following section.

#### Uncertainty in the Information Environment

##### Uncertainty in the Physical Environment

Uncertainty prevails in the physical environment when unknown risks pose potential threats to our societal systems [15,16]. Scholars refer to such threats as “crises,” which can encompass natural disasters, large-scale accidents, social security incidents, and public health emergencies such as the pandemic [37]. Crises are marked by the existence of uncertainty and the imperative for timely decision-making [38]. Therefore, a crucial process during crises is sensemaking. However, the efforts needed for sensemaking will vary as a crisis progresses through stages. The Crisis and Emergency Risk Communication Model delineates 5 common stages in the crisis life cycle, spanning “from risk, to eruption, to clean-up and recovery, and on into evaluation [38].” The eruption of the crisis, also known as the breakout stage, occurs when a key event triggers the crisis [39]. This is the period when the public becomes initially aware of the crisis, characterized by mysteries and heightened motivation to make sense of it. Evidence indicates that the breakout stage of a crisis harbors the highest level of uncertainty and demands extensive sensemaking efforts (eg, government updates [40]; social media communication [41]), consequently leading to a higher incidence of misinformation [42]. This evidence implies that misinformation is more likely to surface and proliferate in tandem with uncertainty in the information environment during the breakout stage compared with other stages throughout a crisis. These insights offer valuable cues for the detection and prediction of misinformation during the COVID-19 pandemic.

##### Uncertainty in the Macro-Media Environment

The macro-media environment encompasses recent media opinions and public attention to trending topics [12]. Governments and mainstream media play a pivotal role in setting the agenda for public attention. During crises such as the COVID-19 pandemic, governments frequently make swift and crucial decisions to safeguard the public. However, these decisions are often made without sufficient transparency, leading to potential uncertainties surrounding their rationale [43]. Such decisions inevitably draw media and public attention, quickly becoming trending topics in mainstream media outlets [44,45]. Regrettably, these rapid decisions often leave audiences with a high level of uncertainty about the reasons behind and the processes involved in making these decisions, potentially paving the way for misinformation. Supporting this notion, Lu [3] identified a correlation between the swift decision to quarantine Wuhan city and the emergence of misinformation regarding government control measures during the early stages of the COVID-19 pandemic in China. The evidence presented indicates that when public attention is directed toward a trending topic that carries uncertainty, misinformation is likely to emerge and spread. In simpler terms, it can be anticipated that when a piece of information is associated with a trending topic characterized by high uncertainty (as opposed to low uncertainty), there is a higher probability that the information could be misinformation and disseminated.

##### Uncertainty in the Micro-Communicative Environment

Differing from the macro-media environment, which offers a macro perspective on what mass audiences have recently read and focused on, the micro-communicative environment provides a micro view of the communication surrounding a specific issue. Both media and individuals tend to communicate using frames or terms imbued with uncertainty when discussing matters that lack evidence or consensus, such as those stemming from emerging science during the COVID-19 pandemic [32,46]. As an illustration, in the initial phase of the pandemic, when Hong Kong officials reported the first instance of a dog testing “weakly positive” for COVID-19 infection, subsequent media reports highlighted that “Hong Kong scientists aren’t sure [emphasis added] if the dog is actually infected or if it picked up the virus from a contaminated surface [47].” Experimental evidence has shown that such uncertainty frames about scientific matters can diminish people’s trust in science [48]. Empirical evidence from real-life social media data further indicates that a communication style marked by ambiguity can potentially lead audiences to generate and disseminate misinformation [32]. This body of findings implies that if information is embedded in uncertain (as opposed to consensus) communication, it is more likely to be misinformation and disseminated.

##### Uncertainty in Message Framing

Uncertainty can also manifest within the message through its framing or word choice. Uncertainty frames are prevalent in misinformation [15,49]. Oh et al [15] illustrated that source ambiguity and content ambiguity are 2 significant features of misinformation. When individuals create a piece of misinformation that lacks evidence and credibility, they often use uncertain words to describe the unreliable source (eg, someone) or the potential rationale (eg, possible, likely) behind the statement. The incorporation of uncertain words can indeed facilitate the spread of misinformation [29,50]. The inclusion of uncertainty expressions in messages leads individuals to perceive the information as more relevant and suitable for themselves [51]. Consequently, if misinformation exhibits a higher level of uncertainty, it is more likely to be accepted and disseminated by the public.

### Research Objectives

Our research objective is to explore whether uncertainty features within the information environment can enhance the effectiveness of misinformation detection and spread prediction. To achieve this, we introduce a novel EUP framework specifically designed for both tasks. We seek to assess the standalone effectiveness of the EUP and anticipate that it can augment the capabilities of existing state-of-the-art misinformation detectors and predictors. Therefore, we conducted experiments to answer the following research questions:

- *Research question 1*: Can EUP be effective in misinformation detection and spread prediction?
- *Research question 2*: Can EUP improve the performances of the state-of-the-art algorithms for misinformation detection and spread prediction?

## Methods

### Overview

**Figure 1:**
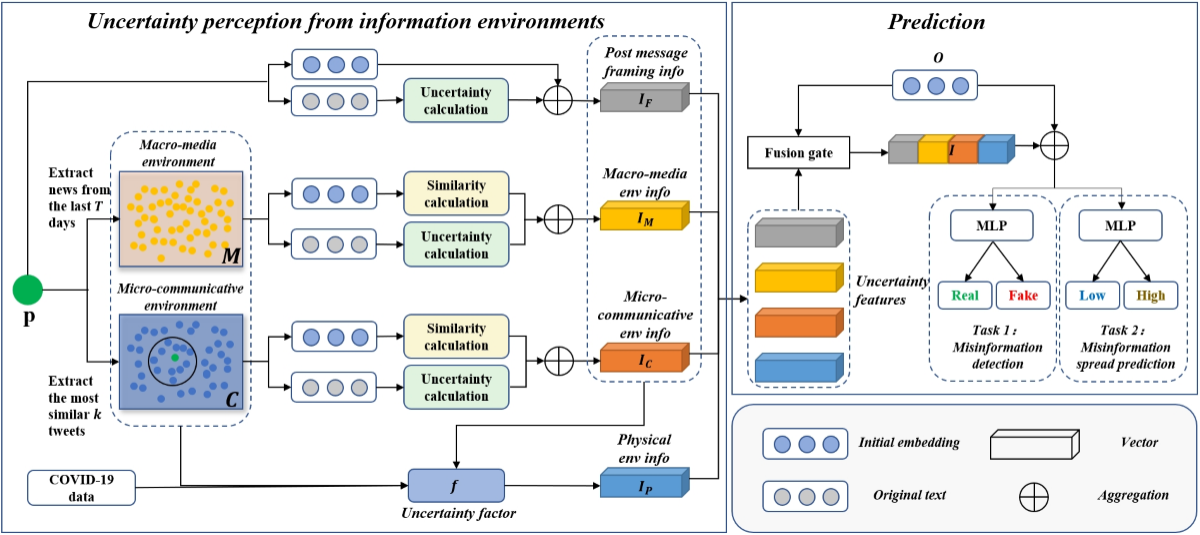
An environmental uncertainty perception (EUP) framework for misinformation detection and spread prediction in the COVID-19 pandemic.

Figure 1 offers an overview of the EUP pipeline. The model consists of 4 uncertainty extraction components. Upon receiving a post (denoted as *p*), the initial step involves constructing its macro-media environment and micro-communicative environment. This is accomplished by extracting recent news and social media data, respectively. Subsequently, we use a probabilistic model and a similarity calculation method to derive the uncertainty information for the 2 environments mentioned above, denoted as *I_M_* and *I_C_*. Likewise, we utilized the probabilistic model to capture the uncertainty of the post *p* itself, resulting in the representation of message framing denoted as *I_F_*. Simultaneously, the operationalization of uncertainty in the physical environment entails using the number of COVID-19 cases and the volume of news as key indicators, denoted as *I_P_*. Lastly, the 4 vectors are integrated using a gate guided by the extracted post feature *o* (which may not necessarily equal *p*) from the misinformation detector, such as bidirectional encoder representations from transformers (BERT) [52]. The fused vectors *I* and *o* are then input into the final classifier, typically a multilayer perceptron (MLP), to predict whether *p* is fake or real in task 1 and low or high in task 2.

### Uncertainty Detection Model

For detecting uncertainty in natural language [53], we used a probabilistic model that considers the local n-gram features of sentences. Each n-gram is assigned a weight that reflects its tendency to convey uncertainty. The definition of each feature involves a quadruplet (type, size, context, and aggregation). “Type” signifies the type of n-gram considered, such as lemma or morphosyntactic pattern. “Size” indicates the size of the n-gram. “Context” serves as an indicator, specifying whether the weight is based on the occurrence frequency of the n-gram in an uncertain sentence or on the occurrence frequency of the n-gram as an uncertainty marker. “Aggregation” refers to the method used to consolidate different scores of the n-grams within a sentence. Multimedia Appendix 1 [49,54-57] furnishes a summary of the diverse features, denoted as *F_i_*, that are scrutinized in the uncertainty detection model.

Next, we exemplify the calculation of uncertainty using 1 of these features, *F*_1_, as an illustration. *F*_1_ is defined by the quadruplet (*Lemma, 1, uncertainty marker,* and *sum*). For each lemma *w*, we can compute the number of occurrences in the corpus, the number of occurrences in uncertain sentences, and the number of occurrences as an uncertainty marker, denoted as *F_s_, F_u_,* and *F_m_*, respectively. The conditional probability of a lemma *w* becoming an uncertainty marker is calculated using the following equation:

*p*(*c*|*w*)=*F_m_*/*F_s_* (1)

where *c* represents the class of context uncertainty under analysis, specifically whether it pertains to being an uncertainty marker. Additionally, we introduce a confidence score linked to the probability of mitigating the impact of instances where certain lemmas occur infrequently in the corpus yet yield a high probability:

conf(*w*)=1–(1–*F_s_*) (2)

*F*_1_ takes into account both the conditional probability of each lemma *w* and the corresponding confidence score in the sentence *s*, and the formula is calculated as follows:



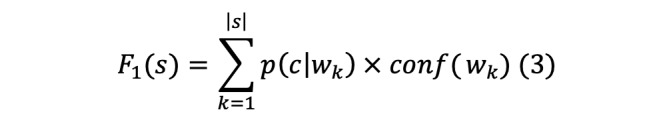



Similarly, other features *F_i_* can be derived using the above method. We generated the uncertainty of the whole sentence by mean pooling to represent the average uncertainty signals of *F_i_*:

*F^A^*^,Mean^ (*s*)=Mean(Norm({*F_i_*(*s*)}^|^*^F^*^|^*_i_*_=1_)) (4)

where Norm(·) denotes the normalization.

### Representation of the Macro-Media Environment

We collect news reports from mainstream media outlets released within *T* days before the post *p* is published to construct a *macro-media environment* according to the following definition:

*M* = {***e***: ***e*** ∈ *E*, 0 ≤ *t_p_* – *t_e_* ≤ *T*} (5)

where *E* denotes the set of all collected news items, *M* denotes the set of news items in the macro-media environment of the post *p*, and *t_p_* and *t_e_* represent the release time of post *p* and news *e*, respectively. For post *p* or each news item *e*, the initial representations are the output of a pretrained language model (eg, BERT [52]), denoted as ***p*** and ***e***, respectively.

The macro-media environment is expected to reflect the impact of a trending topic with high uncertainty on the veracity of a post. That is, if a post is related to a trending topic with (vs without) high uncertainty, it is then expected to be more likely misinformation and disseminated. To this end, the representation of the macro-media environment should consider both the correlation between the post and the environment and the uncertainty of the environment. We first calculate cosine similarity between ***p*** and each news item ***e*** in *E*:

*S*(*p*,*e*) = (*p*·*e*)/(|*p*|·|*e*|) (6)

We combine the similarity and environment representations to represent the similarity representation of a post ***p*** to the environment:



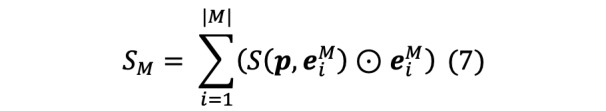



where ***e****^M^_i_* represents each news item in *M* and 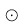 is the Hadamard product operator.

We then measure the uncertainty of the macro-media environment using the model described in the “Uncertainty Detection Model” section. The uncertainty representation of the macro-media environment, denoted as *U_M_*, can be expressed by the following equation:



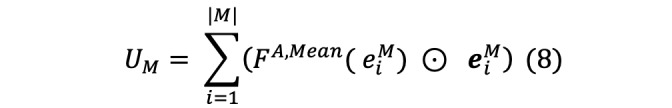



Finally, the macro-media environment of a post *p* is represented as an aggregation of the similarity representation of *p* to the environment (*S_M_*) and the uncertainty representation of the environment (*U_M_*) using an MLP, denoted as *I_M_*:

*I_M_* = MLP(*S_M_*
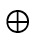
*U_M_*) (9)

where 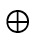 is the concatenation operator. The integration of an MLP is instrumental in the dual objective of retaining crucial information while concurrently achieving data dimensionality reduction. All MLPs are individually parameterized. We omit their index numbers in the above equations for brevity.

### Representation of the Micro-Communicative Environment

We collected tweets from Twitter (X; X Corp.) published within *T* days before the post *p* was published to construct the micro-communicative environment. We calculated the similarity of all tweets to the post *p* and selected the top *k* of them, using them as a *micro-communicative environment* (*C*), which is defined as follows:

C′ = {*v*:*v* ∈ *V*, 0 ≤ *t_p_* – *t_v_* ≤ *T*} (10)

where *V* denotes the set of all collected tweet items and *t_v_* represents the release time of the tweet *v*.

*C* = {*v*: *v* ∈ Top*k*(*p*,*C*′)} (11)

where Top*k*(·) represents the operation of selecting the *k* tweets that have the highest similarity to *p*, *k* = *r*·|*C*′|, and *r* ∈ (0,1) represents the percentage of extraction.

Using the same approach as in the previous 2 sections, we derive the similarity representation of the post *p* to the micro-communicative environment and the uncertainty representation of the environment:



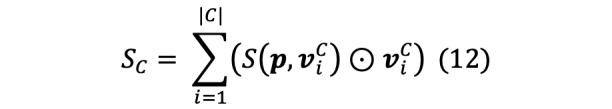





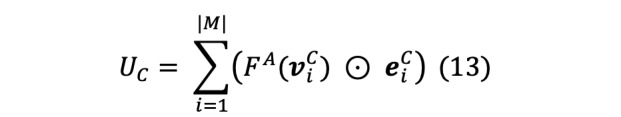



Finally, the micro-communicative environment of a post *p* is represented as an aggregation of the similarity representation of a post *p* to the environment (*S_C_*) and the uncertainty representation of the environment (*U_C_*) using an MLP, denoted as *I_C_*:

*I_C_* = MLP(*S_C_U_C_*) (14)

### Message Framing

To perceive the uncertainty in the message framing of post *p*, we used the same approach as described in the “Uncertainty Detection Model” section to construct the uncertainty representation of the post *p*:

*I_F_*=MLP[***F***(*p*) **
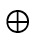
*p***] (15)

### Physical Environment

To measure uncertainty in the physical environment, we collected the daily number of new cases from the start of the COVID-19 outbreak and counted the number of daily news items related to the outbreak, denoted as *N*^Cases^ and *N*^News^, respectively. Intuitively, the higher the number of new cases and news items for a day, the more sensitive the public is to the social environment and the more uncertain the environment is on that day. Thus, the uncertainty factor in the physical environment is defined as follows:

*f^ph^_i_*=Norm(log(1+abs(*N_i_*^Cases^ – *N_i_*_–1_^Cases^)) × log(1+abs(*N*_i_^News^ – *N_i_*_–1_^News^))) (16)

where *f^ph^_i_* denotes the uncertainty factor at day *i* and *abs* is the absolute value operation. For each post, we can obtain the uncertainty factor for its corresponding date *f^ph^*(*p*).

We added the uncertainty factor of the physical environment to the representations of *macro-media environment* (*I_M_*), *micro-communicative environment* (*I_C_*), and *post message framing* (*I_F_*) to get the representation of the physical environment, denoted as *I_P_*:

*I_P_*=(*f^ph^* × I_M_)
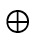
(*f^ph^* × I_C_)
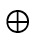
(*f^ph^* × I_F_) (17)

### Prediction

#### Prediction With EUP Alone Without Baseline Models

We concatenate the above 4 environment uncertainty features and feed the result into an MLP layer and a softmax layer for the final prediction:

*I*_EUP_=*I_M_*
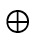
*I_C_*
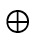
*I_F_*
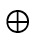
*I_P_* (18)



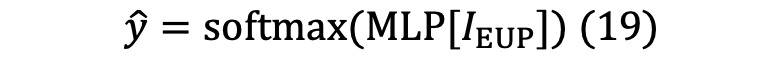



#### Prediction With Baseline Models

We expect that our EUP is compatible with and can empower various misinformation detection and prediction algorithms. Therefore, we used an adaptive feature selection approach based on a gate mechanism to accommodate different misinformation detectors:

*I*=*g_M_*
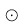
*I_M_* + *g_C_*
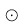
*I_C_* + *g_F_*
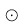
*I_F_* + *g_P_*
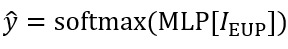
*I_P_* (20)

where ***o*** denotes the last-layer feature from the misinformation baseline algorithm. The gating vector *g_M_*=sigmoid(Linear( ***o*
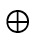
***I_M_*)) and *g_C_*, *g_F_*, and *g_P_* are obtained in the same way. Then, we concatenated ***o*** and *I*,and fed the result into an MLP layer and a softmax layer for the final prediction:



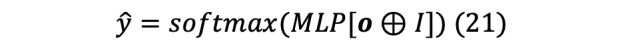



During training, we minimize the cross-entropy loss.

### Ethical Considerations

The study is exempt from ethical review for human subject research for the following reasons. First, the study uses data from 2 publicly available Twitter data sets collected through the official application programming interface (API) of the Twitter platform for gathering tweets. The news data set was obtained from the official websites of news media. Second, the data used in this study are anonymized and do not contain any personally identifiable information. It is also impossible to reidentify individuals from the data set. The data set is stored on a dedicated secure data server, and the analysis is conducted on the platform’s designated site. This process is undertaken for research purposes and adheres to Chinese data privacy laws and regulations. Third, this study does not involve any experimental manipulation of human individuals or other ethical concerns. For instance, it does not include data on children under 18 years of age, which require legally mandated parental or guardian supervision. It also does not encompass sensitive aspects of participants’ behavior or pose any physical, psychological, or economic harm or risk to the research participants.

### Data Set and Experiment

#### Data Set

The statistics and description of our experimental data set are shown in Tables 1 and 2, respectively.

**Table 1:** Statistics of the data set.^a,b^

Data set	Misinformation detection, n			Spread prediction, n			Total, n
	Real	Fake	Low		High		
Train	901	1324	1054		1171	2225	
Value	312	430	360		382	742	
Test	310	432	358		384	742	

^a^News items in *M*=58,095. The corresponding mean and range are 988 and 10-2511, respectively.

^b^Tweet items in *C*=321,656*.* The corresponding mean and range are 793, 138-1214, respectively.

**Table 2:** Descriptions of the data set.

Data	Features	Size, n
Post	Content, created time, retweet count, veracity label, retweeted label	3709
News	Content, created time	58,095
Tweets	Content, created time	321,656

#### Post

We processed and integrated 2 existing COVID-19 data sets, FibVID [58] and CMU_MisCov19 [59], for our experiments. Both data sets have been labeled for veracity by experts, providing ground-truth labels for our experimental evaluations. For FibVID, we extracted data related to COVID-19, assigning veracity tags as 0 (COVID true) or 1 (COVID fake). We relabeled CMU_MisCov19, classifying *calling out or correction, true public health response,* and *true prevention* as *real* tags, and *conspiracy, fake cure, sarcasm or satire, false fact or prevention, fake treatment,* and *false public health response* as *fake* tags. Furthermore, we used the Twitter API to retrieve the number of retweets for all tweets in both data sets. Subsequently, we categorized the retweet labels as low (when the retweet count is 0) and high (when the retweet count is >0) following an analysis of the distribution of retweet numbers. The data revealed that misinformation was predominantly observed from January to July 2020, coinciding with the period of heightened uncertainty during the pandemic outbreak. Consequently, our focus was directed solely to this specific period, resulting in the extraction of 3709 posts from January to July of 2020.

#### Macro-Media Environment

We gathered all the news headlines and brief descriptions from the Huffington Post, NPR, and Daily Mail from January to July 2020, as per the methodology outlined previously [12]. Notably, these 3 outlets represent the left-, center-, and right-wing perspectives, contributing to the diversity of news items for our analysis. We then used the keywords “covid,” “coronavirus,” “pneumonia,” “pandemic,” “epidemic,” “infection,” “prevalence,” and “symptom” to filter these data to ensure that the collected data were relevant to COVID-19. We ended up with 58,095 news items from January to July 2020.

#### Micro-Communicative Environment

We obtained the tweet IDs associated with COVID-19 from an ongoing project [60]. Given the substantial volume, we randomly sampled 1% of these IDs (amounting to approximately 205,581,778 records). Subsequently, using the Twitter API, we retrieved the content associated with these IDs, resulting in a data set comprising 321,656 tweets spanning from January to July 2020.

#### Physical Environment

We compiled the daily count of new worldwide COVID-19 cases starting from January 2020, utilizing the Our World in Data database. Additionally, the daily volume of news data corresponds to the information we gathered during the same period.

### Experimental Setup

#### Tasks

We used the proposed model for 2 tasks:

#### Task 1. Misinformation Detection

The objective was to analyze the text content of a tweet and ascertain whether it contained misinformation.

#### Task 2: Spread Prediction

The objective was to evaluate the text content of a tweet to determine whether it is likely to be retweeted.

#### Uncertainty Features

Following Jean et al [53], we used WikiWeasel [61], a comprehensive corpus consisting of paragraphs extracted from Wikipedia, to compute the frequency of each lemma. The uncertainty score for each sentence is determined using mean pooling *F^A^*^,Mean^. We leverage [62] to acquire sentence representations, relying on pretrained BERT models [52] and subsequent posttraining on news items. In the macro-media environment and the micro-communicative environment, we set *T*=3, *r*=0.1, |*C*|_min_=10.

#### Baseline Models

The baseline models considered are listed in Textbox 1.

**Baseline models.**

1. **Bidirectional long short-term memory**
  Bidirectional long short-term memory (BiLSTM) [63] is a type of recurrent neural network architecture designed for sequence modeling tasks, particularly in natural language processing. It processes input sequences in both forward and backward directions simultaneously, allowing the model to capture information from both past and future contexts.
2. **Event adversarial neural networks**
  Event adversarial neural networks (EANN_T_) [64] is a model using adversarial training to eliminate event-specific features derived from a convolutional neural network for text (ie, TextCNN).
3. **BERT**
  Bidirectional encoder representations from transformers (BERT) [52] is a pretrained language model based on deep bidirectional transformers.
4. **BERT-Emo**
  BERT-Emo [65] is a fake news detection model that integrates multiple sentiment features into BERT.

#### Evaluation Metrics

For both tasks, we used accuracy and macro-*F*_1_-score as evaluation metrics. Additionally, in task 1, we used *F*_1_-scores for fake (*F*_1fake_) and real (*F*_1real_), while in task 2, we considered *F*_1_-scores for low (*F*_1low_) and high (*F*_1high_). Further implementation details can be found in Multimedia Appendix 1.

## Results

### Overview

Tables 3 and 4 showcase the performances of the EUP without baseline models and those of various baseline models, with and without EUP, for the misinformation detection and spread prediction tasks, respectively. The results indicate that the performances of EUP are comparable to those of state-of-the-art baseline models in both tasks. Moreover, it is noteworthy that all baseline models exhibit performance improvements when incorporating EUP for both tasks. These observations suggest the effectiveness of our proposed EUP.

**Table 3:** Model performance comparison on the misinformation detection task without the baseline algorithm or without the EUP^a^ module.^b^

Model	Accuracy	Macro-*F*_1_-score	*F*_1__fake_	*F*_1__real_
EUP	*0.753*	*0.739*	*0.800*	*0.677*
BiLSTM^c^	0.733	0.729	0.783	0.683
BiLSTM + EUP	*0.755*	*0.743*	*0.798*	*0.688*
EANN_T_^d^	0.745	0.730	0.795	0.664
EANN_T_ + EUP	*0.767*	*0.765*	*0.806*	*0.708*
BERT^e^	0.755	0.743	*0.797*	0.689
BERT + EUP	*0.771*	*0.767*	0.796	*0.738*
BERT-Emo	0.749	0.740	0.789	0.691
BERT-Emo + EUP	*0.768*	*0.763*	*0.799*	*0.726*

^a^EUP: Environmental Uncertainty Perception.

^b^The best result in each group is in italics.

^c^BiLSTM: bidirectional long short-term memory.

^d^EANN_T_: event adversarial neural networks.

^e^BERT: bidirectional encoder representations from transformers.

**Table 4:** Model performance comparison on the spread prediction task without the baseline algorithm or without the EUP^a^ module.^b^

Model	Accuracy	Macro-*F*_1_-score	*F*_1__low_	*F*_1__high_
EUP	*0.710*	*0.710*	*0.719*	*0.701*
BiLSTM^c^	0.707	0.705	0.684	0.726
BiLSTM + EUP	*0.734*	*0.733*	*0.738*	*0.729*
EANN_T_^d^	0.717	0.716	0.734	0.698
EANN_T_ + EUP	*0.726*	*0.726*	*0.736*	*0.716*
BERT^e^	0.728	0.728	0.728	0.728
BERT + EUP	*0.743*	*0.743*	*0.752*	*0.734*
BERT-Emo	0.733	0.733	0.730	0.737
BERT-Emo + EUP	*0.741*	*0.741*	*0.733*	*0.749*

^a^EUP: Environmental Uncertainty Perception.

^b^The best result in each group is in italics.

^c^BiLSTM: bidirectional long short-term memory.

^d^EANN_T_: event adversarial neural networks.

^e^BERT: bidirectional encoder representations from transformers.

### Ablation Study

We systematically eliminated individual components, namely, macro-media environment, micro-communicative environment, message framing, and physical environment, and assessed the modeling performances on the data set. Tables 5 and 6 illustrate that, under all experimental conditions, performance degrades when any of these components are removed. These results underscore the effectiveness of all 4 uncertainty features of the information environment for both misinformation detection and spread prediction.

**Table 5:** Ablation study on the misinformation detection task.^a^

Model				Accuracy			Macro-*F*_1_-score		*F*_1__fake_		*F*_1__real_	
**EUP^b^**				*0.753*			*0.739*		0.800		0.677	
		Without *I*_*M*_		0.748	0.738					0.790		*0.687*
		Without *I*_*C*_		0.745	0.720					*0.803*		0.637
		Without *I*_*F*_		0.739	0.734					0.778		0.673
		Without *I*_*P*_		0.747	0.730					0.797		0.663
**BiLSTM^c^ + EUP**				*0.755*			*0.743*		*0.798*		*0.688*	
	Without *I*_*M*_		0.745			0.741		0.793			0.669	
	Without *I*_*C*_		0.741			0.728		0.788			0.668	
	Without *I*_*F*_		0.747			0.735		0.791			0.678	
	Without *I*_*P*_		0.746			0.742		0.796			0.665	
**BERT^d^ + EUP**				*0.771*			*0.767*		*0.796*		*0.738*	
	Without *I*_*M*_		0.762			0.754		0.801			0.707	
	Without *I*_*C*_		0.764			0.761		*0.807*			0.696	
	Without *I*_*F*_		0.761			0.752		0.800			0.705	
	Without *I*_*P*_		0.758			0.751		0.795			0.707	

^a^The best result in each group is in italics.

^b^EUP: Environmental Uncertainty Perception.

^c^BiLSTM: bidirectional long short-term memory.

^d^BERT: bidirectional encoder representations from transformers.

**Table 6:** Ablation study on the spread prediction task.^a^

Model			Accuracy		Macro-*F*_1_-score		*F*_1__low_		*F*_1__high_
**EUP^b^**			*0.710*		*0.710*		0.719		*0.701*
	Without *I*_*M*_	0.697		0.696		0.715		0.676	
	Without *I*_*C*_	0.695		0.694		0.712		0.677	
	Without *I*_*F*_	0.702		0.702		0.714		0.689	
	Without *I*_*P*_	0.708		0.707		*0.721*		0.692	
**BiLSTM^c^ + EUP**			*0.734*		*0.733*		0.738		*0.729*
	Without *I*_*M*_	0.724		0.723		0.735		0.711	
	Without *I*_*C*_	0.721		0.721		0.716		0.726	
	Without *I*_*F*_	0.717		0.716		0.731		0.702	
	Without *I*_*P*_	0.726		0.723		*0.753*		0.693	
**BERT^d^ + EUP**			*0.743*		*0.743*		0.752		*0.734*
	Without *I*_*M*_	0.741		0.739		0.764		0.713	
	Without *I*_*C*_	0.741		0.738		*0.766*		0.711	
	Without *I*_*F*_	0.736		0.735		0.753		0.716	
	Without *I*_*P*_	0.740		0.738		0.759		0.717	

^a^The best result in each group is in italics.

^b^EUP: Environmental Uncertainty Perception.

^c^BiLSTM: bidirectional long short-term memory.

^d^BERT: bidirectional encoder representations from transformers.

### The Effect of the Day Parameter T

**Figure 2:**
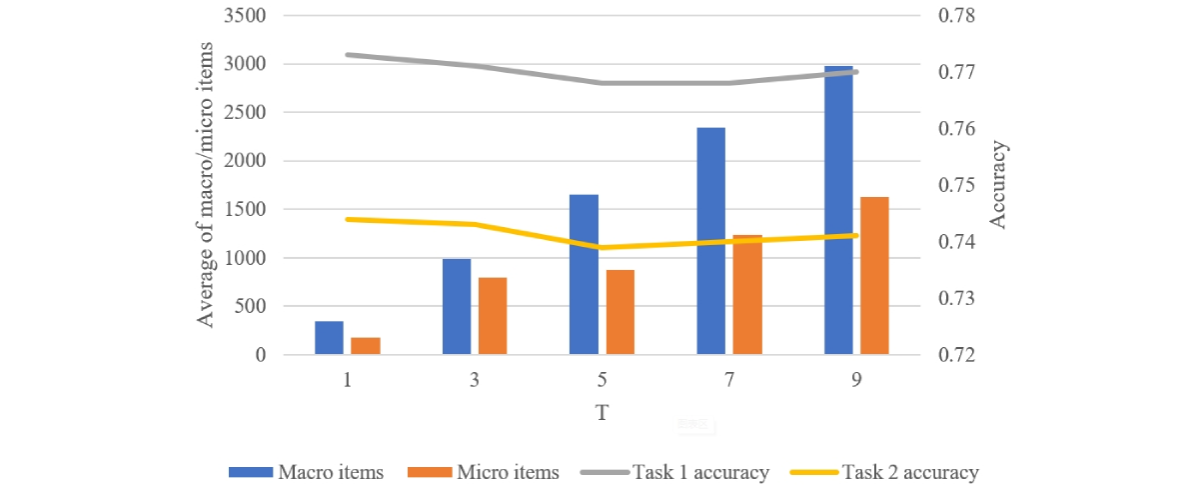
The effect of the day parameter T. Lines show the accuracies of both tasks and bars show the average number of news and tweet items in the environments.

To explore the impact of the day parameter (*T*) on the results during the construction of the macro-media environment and the micro-communicative environment, we experimented with different values of *T*. Specifically, we sequentially set *T*=1, 3, 5, 7, and 9 for the BERT + EUP model, and the experimental results are depicted in Figure 2. Despite the fact that increasing *T* results in larger macro-media and micro-communicative environments, the optimal performance was achieved when *T*=1.

### The Effect of the Rate Parameter r

**Figure 3:**
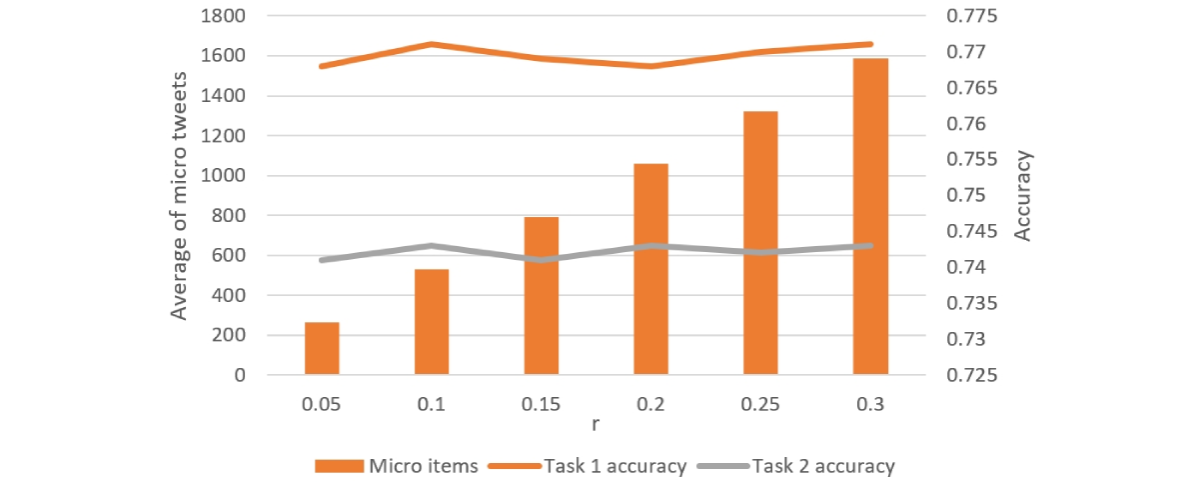
The effect of the rate parameter r. Lines show the accuracies of both tasks and bars show the average number of tweet items in the environment.

We maintained the setting *T*=3 and systematically varied *r*, using values of 0.05, 0.1, 0.15, 0.2, 0.25, and 0.3 on the BERT + EUP model to examine the impact of *r* on the experimental results, as illustrated in Figure 3. The accuracy performance exhibited fluctuations with varying values of *r*. Notably, the highest accuracy for both tasks was observed when *r*=0.1.

### Evaluation on Imbalanced Data

**Figure 4:**
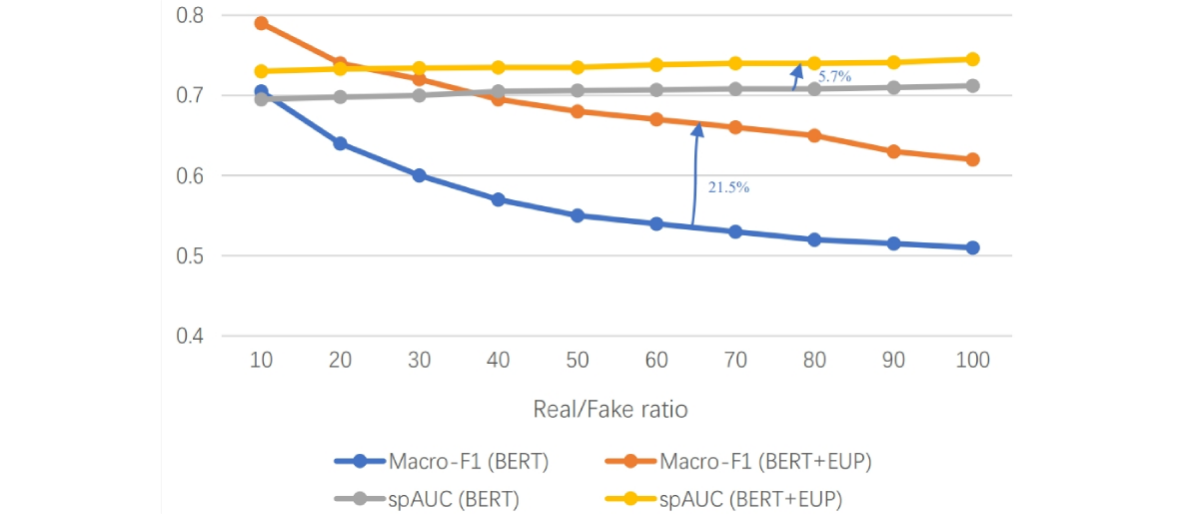
Performance of macroF1 and spAUC values across datasets with varying ratios.

In real-world scenarios, the distribution of real and fake information often exhibits significant imbalance. To evaluate the efficacy of our proposed EUP framework on unbalanced data sets, we conducted tests on data sets with varying ratios of real to fake data, ranging from 10:1 to 100:1. We measured and reported macro-*F*_1_-scores and standardized partial area under the curve (AUC) with a false-positive rate of at most 0.1 (ie, spAUCFPR≤0.1 [66]) to assess the effectiveness of our EUP framework in handling nonbalanced data sets. As depicted in Figure 4, EUP yields relative improvements of 21.5% and 5.7% in macro-*F*_1_-score and spAUCFPR≤0.1, demonstrating its effectiveness on unbalanced data sets.

## Discussion

### Principal Findings

First, this study enhances scholars’ comprehension of the misinformation detection and spread prediction problem by highlighting the significance of uncertainty in information environments. Notably, this research contributes to the literature by recognizing uncertainty features in the information environments of misinformation as a pivotal factor for improving detection and prediction algorithms during a pandemic. Our findings underscore that the EUP alone is sufficient for both tasks and has the potential to enhance the capabilities of state-of-the-art algorithms. In contrast to prior misinformation research that primarily concentrates on post content (such as post theme, sentiments, and linguistic characteristics, as seen in [6,11,29]) and network connections (eg, number of followers [25]) on social media, this study advances scholars’ understanding of the misinformation problem by emphasizing the importance of uncertainty in information environments. Recognizing and incorporating uncertainty as a fundamental concept in misinformation detection and spread prediction during crises hold theoretical significance. This is particularly relevant as a crisis is characterized by its unpredictable, unexpected, and nonroutine nature, inherently giving rise to uncertainty [38,67]. This uncertainty has been theorized to compel individuals to seek information as a coping mechanism for dealing with the anxiety and pressure generated by uncertainty. This process allows people to diminish uncertainty, restore a sense of normalcy, and alleviate anxiety [14,68]. Regrettably, this coping mechanism can inadvertently fuel the proliferation and dissemination of misinformation, particularly when there is a lack of timely and accurate information, contributing to the concurrent occurrence of an infodemic [6,11,50]. The current research seeks to advance the literature by establishing the legitimacy of uncertainty in the information environments of misinformation as a central indicator for the detection and prediction of misinformation during public health crises.

Second, this study delves into the intricacies of uncertain information environments for misinformation across 4 distinct scales, namely, the physical environment, macro-media environment, micro-communicative environment, and message framing. Our findings demonstrate the effectiveness of all 4 uncertainty features in misinformation detection and spread prediction. In contrast to prior misinformation literature during the COVID-19 pandemic, which often overlooked the role of the information environment in increasing the likelihood of misinformation dissemination, our research emphasizes the importance of considering uncertainty beyond the content of misinformation itself, such as ambiguous wording [29,50]. Our study broadens the concept of linguistic uncertainty in misinformation message framing to encompass a more comprehensive uncertainty across various information environments. We define uncertainty in information environments using a multiscale approach that highlights the significance of the interaction between the physical environment and macro-/micro-media environments. This approach diverges from focusing on a single dimension, such as ambiguities about official guidelines and news reports [18], or the misinformation framing strategy on social media [29].

Third, our findings indicate that uncertainties in information environments play a crucial role as motivators for the emergence and spread of misinformation. While previous studies have provided preliminary evidence suggesting that uncertainty stemming from government policies and news media could coincide with the occurrence of related misinformation during the COVID-19 pandemic, often relying on descriptive big data analyses [3,32], our study contributes stronger empirical evidence. We leverage machine learning techniques to demonstrate that uncertainty arising from the crisis and crisis communication through media can indeed incentivize individuals to generate and disseminate misinformation. Significantly, our findings revealed that the algorithm achieved its best performance for both detection and spread prediction tasks when incorporating items from the information environments published 1 day before the post (*T*=1). This discovery emphasizes the acute impact of uncertainty in the information environment on the emergence and spread of misinformation, underscoring the importance of timely uncertainty reduction in crisis communication. Furthermore, the algorithm attained the highest accuracies when it included items highly relevant to the post but with an appropriate size (*r*=0.1). This rationale is reasonable, as a too-small *r* may fail to encompass enough misinformation-related items, while a larger *r* might include a significant amount of irrelevant information. The evidence theoretically establishes a connection between crisis communication research and misinformation research, reinforcing the notion that crisis communication and misinformation containment are 2 intertwined aspects of crisis management [3].

This study offers significant practical implications for misinformation detection and spread prediction. First, unlike previous studies that separately investigated computational frameworks for these tasks [24,29], this study introduces a unified uncertainty–based framework capable of addressing both tasks simultaneously. Second, our framework operates instantaneously, as it only requires easily accessible data such as posts, mainstream news, and relevant social media discussions published a few days prior. Moreover, the uncertainty detection algorithm has been trained using external data, rendering our algorithm easy to implement and capable of providing timely detection and prediction for streaming textual data. Third, this study affirms the effectiveness of uncertainty in various information environments for detecting and predicting misinformation on social media. Hence, the 4 proposed uncertainty components in information environments could be leveraged by social media platforms to improve the accuracy of misinformation detection and spread prediction, thereby safeguarding individuals from harm caused by infodemic. The benefits offered by our algorithm may serve as an impetus for integrating uncertainty components into practical systems.

### Limitations and Future Work

This study is the first to incorporate the uncertainty present in the information environment of a post for both misinformation detection and spread prediction. However, it has some limitations. First, our framework concentrated solely on text-only detection and prediction. Future work should extend the framework to incorporate multimodal and social graph–based detection. Second, we used an uncertainty detection algorithm developed from a generic corpus sourced from Wikipedia. Nevertheless, past research has indicated that expressions of uncertainty may vary slightly across domains [53]. In other words, uncertainty expressions in the context of the COVID-19 pandemic may differ from those in general situations. Therefore, future work should aim to enhance our uncertainty measure by utilizing a corpus specifically designed for uncertainty detection in the discourse related to COVID-19.

### Conclusions

We introduced an EUP framework for both misinformation detection and spread prediction. Our framework delves into uncertainty within information environments across 4 scales: the physical environment, macro-media environment, micro-communicative environment, and message framing. The experiments demonstrated the effectiveness of our proposed uncertainty components in enhancing the performance of existing models. There are several directions for further investigation and extension of this work. First, we can explore the impact of different news and social media environments (eg, biased vs neutral; left wing vs right wing) on the emergence and spread of misinformation. Second, extending our algorithms to include multimodal misinformation detection could be beneficial, as misinformation increasingly incorporates images and videos. Third, investigating the interaction between misinformation detection and spread prediction using a multitask, transfer-learning model is a promising avenue, given the shared uncertainty framework identified in this study for both tasks.

## Appendix

Multimedia Appendix 1 Uncertainty features.

## Abbreviations

API: application programming interface
AUC: area under the curve
BERT: bidirectional encoder representations from transformers
BiLSTM: bidirectional long short-term memory
EANNT: event adversarial neural networks
EUP: Environmental Uncertainty Perception
MLP: multilayer perceptron
spAUCFPR: standardized partial area under the curve with a false-positive rate
TextCNN: convolutional neural network for text

## Data Availability

The data sets generated and analyzed during this study are available from the corresponding author on reasonable request.
