# Supplementary material for: An Environmental Uncertainty Perception Framework for Misinformation Detection and Spread Prediction in the COVID-19 Pandemic: Artificial Intelligence Approach
Source: JMIR AI. 2024 Jan 29;3:e47240. doi: 10.2196/47240 (PMC11041461; doi:10.2196/47240)
Supplement: Multimedia Appendix 1 [file ai_v3i1e47240_app1.docx]

1. ***Uncertainty features***

Table 1a shows all features used in uncertainty detection.

| **Table 1a**  All features used in uncertainty detection. | | | | |
| --- | --- | --- | --- | --- |
| Features | Type | Size | Context | Aggregation |
| $F_{1}$ | Lemma | 1 | uncertainty marker | sum |
| $F_{2}$ | Lemma | 2 | uncertainty marker | sum |
| $F_{3}$ | Lemma | 1 | ∈ uncertain sentence | sum |
| $F_{4}$ | $PoS$ | 5 | ∈ uncertain sentence | sum |
| $F_{5}$ | Lemma | 1 | ∈ uncertain sentence | max |

1. **Implementation Details**

We implemented our approach using the Pytorch framework[64]. Parameters were optimized using Adam’s algorithm[65]. We split the dataset into a training set, a validation set, and a test set, with a split ratio of 6:2:2 and no overlap. We reported test results based on the trained parameter settings of the best validation epoch. We run all our experiments on a single NVIDIA GeForce RTX 2080Ti.

**Implementation of Base Models**

- **BiLSTM:** We use the glove.840B.300d13 [66] embedding to represent each token in a sentence and get 128-dimensional contextual sentence features with a single-layer Bi-LSTM encoder. The hidden dim is set to 128.
- **EANN_T_:** We use the text-only variant of the original EANN. The word embeddings and hidden dim are the same as Bi-LSTM. The kernel sizes for both datasets are [1, 2, 3, 4]. The number of filters is 30. We ran K-means[67] in the scikit-learn package to gather the training samples into 300 clusters (corresponding to 300 events).
- **BERT:** We use BERT-based-uncased to tokenize the texts. The maximum length is 128. The dimension of each token representation is 768.
- **BERT-Emo:** We use the publisher-emotion-only variant in [49] that excludes the social emotion features. Other parameters are the same as for BERT.

1. **Calculation of spAUC**

We use the standardized partial AUC (spAUC) (McClish, 1989) for evaluation. It is suitable to our scenario where we expect the method to find fake news posts as many as possible with an acceptable misclassification rate of real ones. The partial AUC over the false positive rate [0, x] is:

$$\mathrm{pAUC}_{\mathrm{FPR}\leq x}=\int_{0}^{x} \mathrm{ROC}(x)dx$$

where ROC is the Receiver Operating Characteristic curve. The spAUC is calculated as

$$\mathrm{spAUC}_{\mathrm{FPR}\leq x}=\frac{1}{2}\left( 1+\frac{\mathrm{pAUC}_{\mathrm{FPR}\leq x}-\frac{1}{2}x^{2}}{x-\frac{1}{2}x^{2}} \right)$$

In our experiment, we use the implementation in the scikit-learn package.
